# Characterizing Electrocochleography in Cochlear Implant Recipients with Residual Low-Frequency Hearing

**DOI:** 10.3389/fnins.2017.00141

**Published:** 2017-03-23

**Authors:** Christofer W. Bester, Luke Campbell, Adrian Dragovic, Aaron Collins, Stephen J. O'Leary

**Affiliations:** ^1^Department of Otolaryngology, University of MelbourneMelbourne, VIC, Australia; ^2^Royal Victorian Eye and Ear HospitalMelbourne, VIC, Australia

**Keywords:** cochlea, cochlear implant, cochlear microphonic, electrocochleography, hearing loss

## Abstract

**Objective:** Lay the groundwork for using electrocochleography (ECochG) as a measure of cochlear health, by characterizing typical patterns of the ECochG response observed across the electrode array in cochlear implant recipients with residual hearing.

**Methods:** ECochG was measured immediately after electrode insertion in 45 cochlear implant recipients with residual hearing. The Cochlear Response Telemetry system was used to record ECochG across the electrode array, in response to 100- or 110-dB SPL pure tones at 0.5-kHz, presented at 14 per second and with alternating polarities. Hair cell activity, as the cochlear microphonic (CM), was estimated by taking the difference (DIF) of the two polarities. Neural activity, as the auditory nerve neurophonic (ANN), was estimated by taking the sum (SUM) of the two polarities. Prior work in humans and animal studies suggested that the expected ECochG pattern in response to a 0.5-kHz pure tone is an apical-peak in CM amplitude and latency.

**Results:** The most prevalent pattern was a peak in the DIF amplitude near the most apical electrode, with a prolongation of latency toward the electrode tip; this was found in 21/39 individuals with successful ECochG recordings. The 21 apical-peak recipients had the best low-frequency hearing. A low amplitude, long-latency DIF response that remained relatively constant across the electrode array was found in 10/39 individuals, in a group with the poorest low- and high-frequency hearing. A third, previously undescribed, pattern occurred in 8/39 participants, with mid-electrode peaks in DIF amplitude. These recipients had the best high-frequency hearing and a progressive prolongation of DIF latency around the mid-electrode peaks consistent with the presence of discrete populations of hair cells.

**Conclusions:** The presence of distinct patterns of the ECochG response with relationships to pre-operative hearing levels supports the notion that ECochG across the electrode array functions as a measure of cochlear health.

## Introduction

Cochlear implants (CIs) are no longer restricted to individuals with severe-to-profound hearing loss. Instead, many implant recipients have substantial levels of low-frequency residual hearing, and a goal of modern implant designs and surgical techniques is to preserve this hearing for electro-acoustic stimulation (EAS; Gantz et al., 2005). Efforts to combine residual hearing and EAS have been hampered by the absence of methods to map the function of neurosensory elements along the cochlea. Such a map would identify frequencies that are appropriate for acoustic stimulation and those that require electrical stimulation, identified as an important factor in the success of this combined delivery method (Gantz and Turner, 2004). Here we demonstrate that this can be achieved with the direct recording of electrocochleography (ECochG) along the length of a cochlear implant electrode.

ECochG has recently become available using intra-cochlear electrodes in CI recipients (Calloway et al., 2014; Campbell et al., 2015; Dalbert et al., 2015). It is a cochlear potential derived from neural and sensory sources in response to transient acoustic stimuli presented with alternating polarity. A frequency-following hair cell response known as the cochlear microphonic (CM) is derived by taking the difference of the two alternating responses (DIF) (Ruben et al., 1961; Dallos, 1973; Patuzzi et al., 1989). ECochG also contains the phase-locked neural response of the auditory nerve as the auditory nerve neurophonic (ANN). As phase-locking occurs preferentially as inner hair cells depolarize (Palmer and Russell, 1986), it results in distortions in the ECochG trace that occur at even harmonics of the acoustic input. Therefore, the ANN is derived by summing the alternating phase responses (SUM), and isolating the 2nd harmonic of the stimulus frequency (Weinberger et al., 1970). It is important to note that the DIF trace, while dominated by the CM, will contain some neural response as demonstrated by Forgues et al. (2014). Similarly, while the SUM trace is dominated by the ANN, at the high sound intensities required for ECochG in CI recipients this response may include some hair cell activity due to asymmetric saturation in the input-output function of the hair cell response (Teich et al., 1989).

ECochG has been recorded from intracochlear electrodes in hearing animals responding to pure acoustic tones. As the site of recording progresses from the base of the cochlea toward the location where the cochlea is most sensitive to the stimulus, there is an exponential increase in CM amplitude and prolongation of its latency. The CM amplitude decreases rapidly at cochlear sites apical to this “characteristic” frequency (Honrubia and Ward, 1968). There have been three previous reports of ECochG recorded from multiple locations along the human cochlea (Calloway et al., 2014; Dalbert et al., 2015; Campbell et al., 2016). Dalbert et al. (2015) recorded ECochG from multiple electrodes along a mid-scalar electrode array (HiFocus Mid-Scala electrode, Advanced Bionics, USA), 12 or more weeks after implantation. Contrary to Calloway et al. (2014), all eight participants exhibited ECochG responses with relatively constant amplitude across the array, or showed a peak in the CM response on basal electrodes in response to 0.5- or 1-kHz tones. It was suggested that the unexpectedly flat responses and basal-peak responses arose from the proximity of the electrode to the auditory nerve, or the influence of intra-scalar fibrosis on the current path in the vicinity of the electrode (also suggested by Campbell et al., 2015). In contrast, Formeister et al. (2015) made recordings at multiple insertion depths from a single recording electrode on a flexible carrier that was inserted into the cochlea during surgery, just prior to implantation of the commercial CI. These investigators found the relationship seen in the previous animal experiments of Honrubia and Ward (1968), with five of eight patients exhibiting a rise in CM amplitude as depth increased, in response to a 0.5-kHz tone. We have made similar observations when recording ECochG from the apical-most electrode of an implant manufactured by Cochlear Ltd during its insertion into the cochlea (Campbell et al., 2016). Whether the difference in response patterns observed between these studies reflects differences in the time between implantation and recording, differences in residual hearing between CI recipients, or the intracochlear position of the recording electrode between devices remains unclear.

In the present work intracochlear ECochG was recorded across the electrode array during surgery, immediately after insertion of the electrode array, in 45 CI recipients who received Cochlear's CI422 or 522 implants. These devices have a thin, flexible electrode that traverses the lateral wall of the cochlea. The aim of the present work was to characterize patterns of ECochG across the electrode array and relate these patterns of response to pre-operative hearing levels. We predicted that there would be a restricted number of patterns of ECochG response, and that these would be associated with the shape of the pre-operative audiogram.

## Methods

### Clinical information

Forty-five adults who received a CI422 or CI522 cochlear implant (Cochlear Ltd, Sydney, Australia) with the “Slim Straight” electrode array had ECochG recordings made from the electrode array immediately after its insertion. All participants had pre-operative hearing thresholds lower than 100-dB HL at 0.5-kHz and a post-lingual hearing loss.

The CI422 and CI522 implants have Cochlear's Slim Straight electrode, an array with 22 half-band intra-cochlear electrodes. The cochlea was approached via a posterior tympanotomy, and the electrode inserted through an incision made in the round window to a depth of between 20- and 25-mm, at the surgeon's discretion. All participants had a full insertion of at least 20-mm, confirmed at the time of surgery and with a post-operative cone-beam CT scan.

This research was conducted under the auspices of the Human Research and Ethics Committee of the Royal Victorian Eye and Ear Hospital HREC (#14/1171H). All patients provided informed, written consent for their participation in the study, and for its dissemination through publication.

### Equipment and information processing

Electrocochleography was recorded using the Cochlear Response Telemetry (CRT) system previously described by the investigators (Campbell et al., 2015, 2016). Acoustic stimuli were generated digitally using a USB data acquisition card (DT9847, Data Translation, USA), and presented using an ER3A insert earphone (Etymotics, USA). The acoustic stimuli were 12-ms in length with 1-ms linear onset and offset ramps and a 50-ms inter-stimulus interval. Alternating rarefaction and condensation phases were presented, and stored separately. The intensity of the acoustic stimuli was calibrated with peak-to-peak amplitudes equal to the dB HL scale for insert earphones (ISO 389-2:1994).

The CRT system uses the implant's Neural Response Telemetry™ (NRT) amplifier to record from the intra-cochlear CI electrodes. These recordings are made between any one of the intra-cochlear electrodes and the extra-cochlear plate electrode located on the body of the implant. Recording windows were 20-ms in duration, digitized at 20-kHz and streamed to a Dell laptop (Dell, USA), via a Cochlear Freedom™ programming POD. Each ECochG waveform is an average of 100 presentations. The stimuli and recording were coordinated by in-house custom-written software, which interfaced with the Freedom™ sound processor using the Cochlear Device Interface (CDI) libraries (4.15.02). ECochG was recorded from the most apical electrode (22) and then every second electrode until the second most basal electrode. In this study, ECochG was characterized across the electrode array in response to a 0.5-kHz tone pip, delivered at either 100- for patients with ≤ 70 dB HL at 0.5-kHz or 110-dB for those with >70 dB HL. The 0.5-kHz stimulus frequency was chosen as the closest frequency apical to the average angular insertion depth in a CI422/CI522 patient (410°, or ~0.75-kHz, O'Connell et al., 2016) for which audiometric thresholds are routinely measured. The 1-ms linear onset and offset ramps will result in a loss of frequency specificity (Skinner and Jones, 1968), calculated by FFT to be a broadening of the stimulus by 0.075-kHz either side of 0.5-kHz starting at −20 dB to the peak at 0.5-kHz. Frequency specificity is already decreased due to the high sound pressure level used (Russell and Nilsen, 1997), and considerable sensorineural hearing loss present in the cohort (Gummer and Johnstone, 1984).

To estimate the CM and ANN components of the ECochG waveform, the recordings were processed by either adding the alternating phases responses (SUM) to estimate the ANN or by subtracting them (DIF) to estimate the CM (Adunka et al., 2006; Choudhury et al., 2012; Campbell et al., 2015). To isolate the magnitude of the stimulus frequency-matching CM in the DIF trace, the magnitude at the stimulus frequency was calculated by Fast Fourier Transform (FFT). For the ANN, the asymmetric neural saturating response in the SUM trace was isolated using the FFT magnitude at the 2nd harmonic. The latency of these responses was measured by calculating the FFT phase difference from the response at the most basal electrode to each successive electrode, which used the 2nd harmonic of the SUM trace or the 1st harmonic of the DIF trace. A noise floor for each trace was calculated from FFT bins ± 2 from the frequency of interest, where each FFT bin was 62.5 Hz wide, and ECochG responses were considered robust if the amplitude exceeded the calculated noise floor plus 3 standard deviations.

The absolute latency of the DIF response was measured as the first deflection from baseline after the first pressure change in the ear canal (calibrated using a Bruel and Kjaer ½ inch microphone, oscilloscope, and 2cc coupler) from either the most basal or most apical electrode.

An electrode was considered to have a CM peak if its magnitude was >30% above the mean magnitude across the electrode array. If more than one electrode satisfied this condition, then the electrode with the largest CM was considered the peak. Multiple peaks were recorded if there was an electrode with a greater than 30% drop between one peak and the next more apical electrode.

## Results

**Figure 1:**
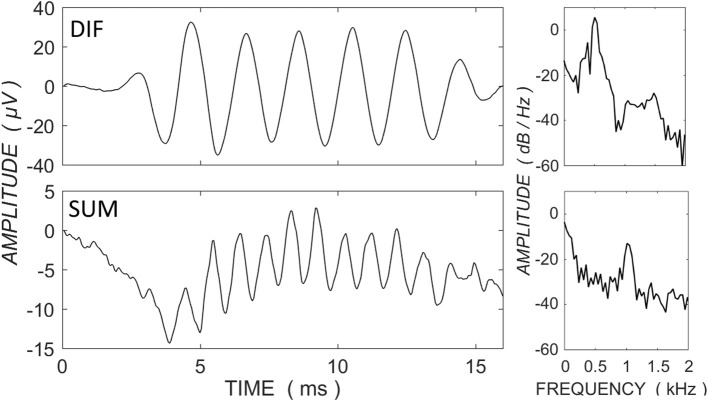
**ECochG traces for the difference (DIF - upper panels) and sum (SUM - lower panels) responses in a single CI recipient with <70 dB HL at 0.5-kHz and in response to a 0.5-kHz tone burst at 100 dB HL**. Power spectral density functions are shown to the right of the traces (expressed as dB relative to 1 μV). The primary power for the DIF trace is concentrated at the fundamental frequency, consistent with a contribution primarily by the frequency-matching hair cell response, whereas the power in the SUM trace is concentrated at the secondary harmonic, consistent with the neural saturating response.

Electrocochleography could be recorded across the electrode array in response to 0.5-kHz tone in all but six participants, in whom there was no detectable ECochG response on any electrode. Figure 1 shows example DIF and SUM traces, with power spectral density functions from a single CI recipient with <60 dB HL at 0.5-kHz. Figure 1 demonstrates that the bulk of response power in the DIF trace is located at the stimulus frequency, consistent with a primary contribution by the CM, whereas the power in the SUM trace is concentrated at the secondary harmonic, consistent with asymmetric saturation in the neural response.

Median hearing level for all participants who showed a detectable ECochG was 60-, 65-, 85-, 100-, and 110-dB HL at 0.25-, 0.5-, 1-, 2-, and 4-kHz.

### Apical peak

**Figure 2:**
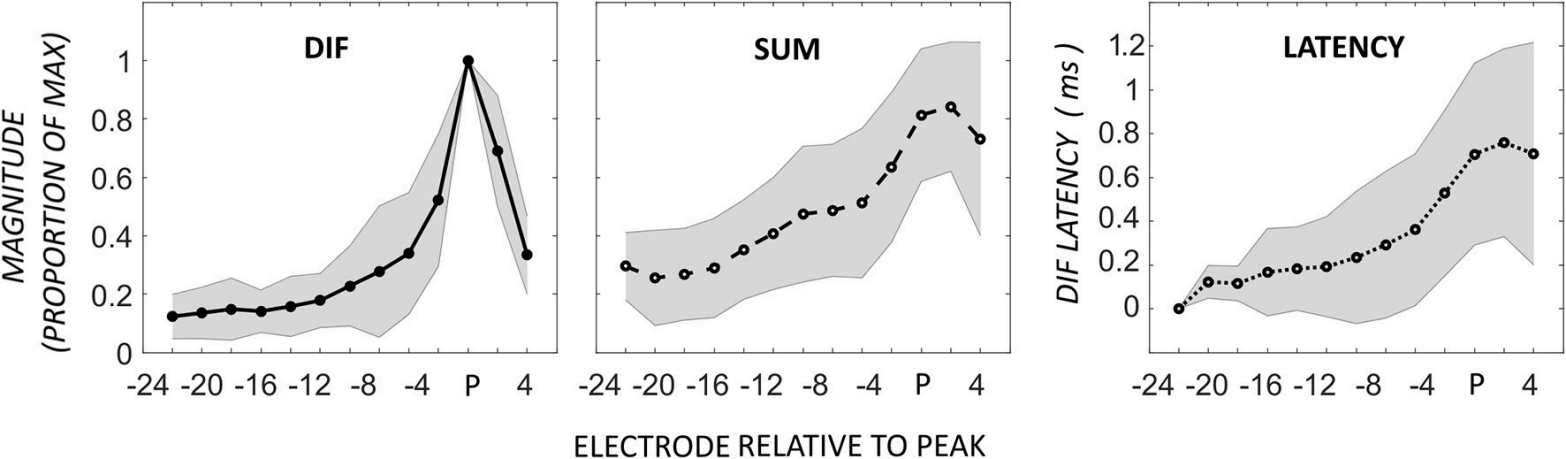
**ECochG responses in 21 patients with the maximum DIF amplitude at apical electrodes**. Responses here have been normalized to the maximum response in each individual, as well as to the electrode with the peak DIF response (P). The amplitudes of the SUM response are reported normalized to the electrode with the peak DIF response. The DIF latency is reported relative to the response on the most basal electrode in each individual. Shaded area represents ± 1 *SD*.

The acoustic stimulus was a 0.5-kHz tone, and ECochG was recorded from 11 electrodes across the array. The most prevalent response pattern (21 participants) was a growth of the DIF amplitude to a peak near the apical tip of the electrode, defined as a single CM peak on the most apical 6 electrodes located proximal to the 0.5-kHz characteristic frequency region in the cochlea. The major acoustic generator contributing to this response is the CM (Dallos, 1973; Patuzzi et al., 1989). The DIF and SUM amplitudes, and DIF latencies are shown relative to the electrode with the peak DIF amplitude in Figure 2. In this figure, the amplitude of the responses has been normalized relative to the peak amplitude in the respective individual. The mean absolute DIF amplitude on electrodes basal to the peak DIF responses was 3.4 μV ± 0.3 SEM. The mean maximum absolute DIF amplitude on apical peaks was 22.1 μV ± 5.6 SEM. In these participants, the peak DIF amplitude was located at one of the more apical electrodes, specifically on electrodes 22 (i.e., at the tip, *n* = 9), 20 (*n* = 6), 18 (*n* = 4), or 16 (*n* = 2). A rapid increase in DIF amplitude was found up to the electrode exhibiting the peak, with a comparably rapid drop off in amplitude once that at more apical electrodes. The SUM response showed a similar pattern with gradual increase in amplitude that reached its maximum at, or slightly above, the peak electrode. The SUM response is largely derived from the frequency-following potential of the auditory nerve, the ANN (Weinberger et al., 1970). Across the group, there was a moderate to strong positive correlation between DIF and SUM amplitudes (Pearson product-moment correlation coefficient, mean *r* = 0.68, ranging from 0.18 to 0.94). While this correlation was strong, the peak SUM response was on a more apical electrode than the peak DIF response in the majority of patients (*n* = 12), and less frequently on the same (*n* = 7), or a more basal electrode (*n* = 2). In this group, absolute latency increased across the electrode array from 1.22-ms ± 0.67 until the peak was reached (2.40-ms ± 0.66) and there was a strong, positive correlation between the DIF amplitudes and latencies (Pearson product-moment correlation coefficient mean *r* = 0.76, ranging from 0.45 to 0.96).

### Flat-response

**Figure 3:**
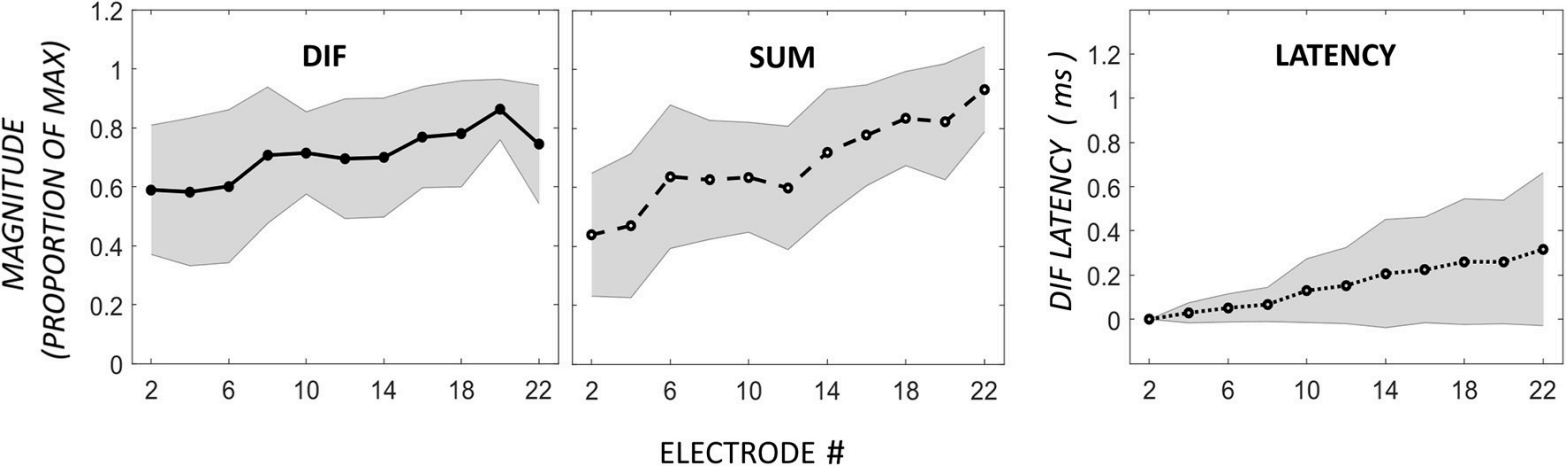
**Comparison of DIF, SUM, and DIF latency responses in 10 participants with a flat response across electrodes 2–22**. Note that the horizontal axis in this figure is not normalized to the maximum DIF response as in Figure 2, as these subjects showed a flat response pattern without a distinct maximum.

In addition to the pattern of ECochG with an apical peak in DIF amplitude, we observed a pattern of flat DIF amplitudes across the electrode array in 10 participants, defined as individuals with no detected CM peaks. The DIF and SUM amplitudes, and DIF latencies are shown across the electrode array in this group in Figure 3.

In this group, there was no apical rise in DIF amplitudes proximal to the 0.5-kHz region in the cochlea. However, absolute DIF amplitues across the whole electrode array in the flat-responders were not significantly different to the responses across the basal electrodes in the apical-peak group (means of 3.4 ± 6.5 and 1.3 ± 0.4 μV for the apical-peak and flat-response groups respectively, all responses passed 1-sample Kolmogorov–Smirnov tests for normality). The flat-responders showed a gradual increase in SUM amplitude with increasing electrode depth, and there was a weak relationship between DIF and SUM amplitudes (Pearson product-moment correlation coefficient mean *r* = 0.36, with a range from −0.63 to 0.92). The latency of the DIF response rose from 2.31-ms ± 1.1 on the most basal electrode to 2.6-ms ± 1.2 at the most apical. The latency on the most basal electrode in this group was comparable to that recorded from the tip electrodes in the apical-peak group.

### Mid-electrode peaks

**Figure 4:**
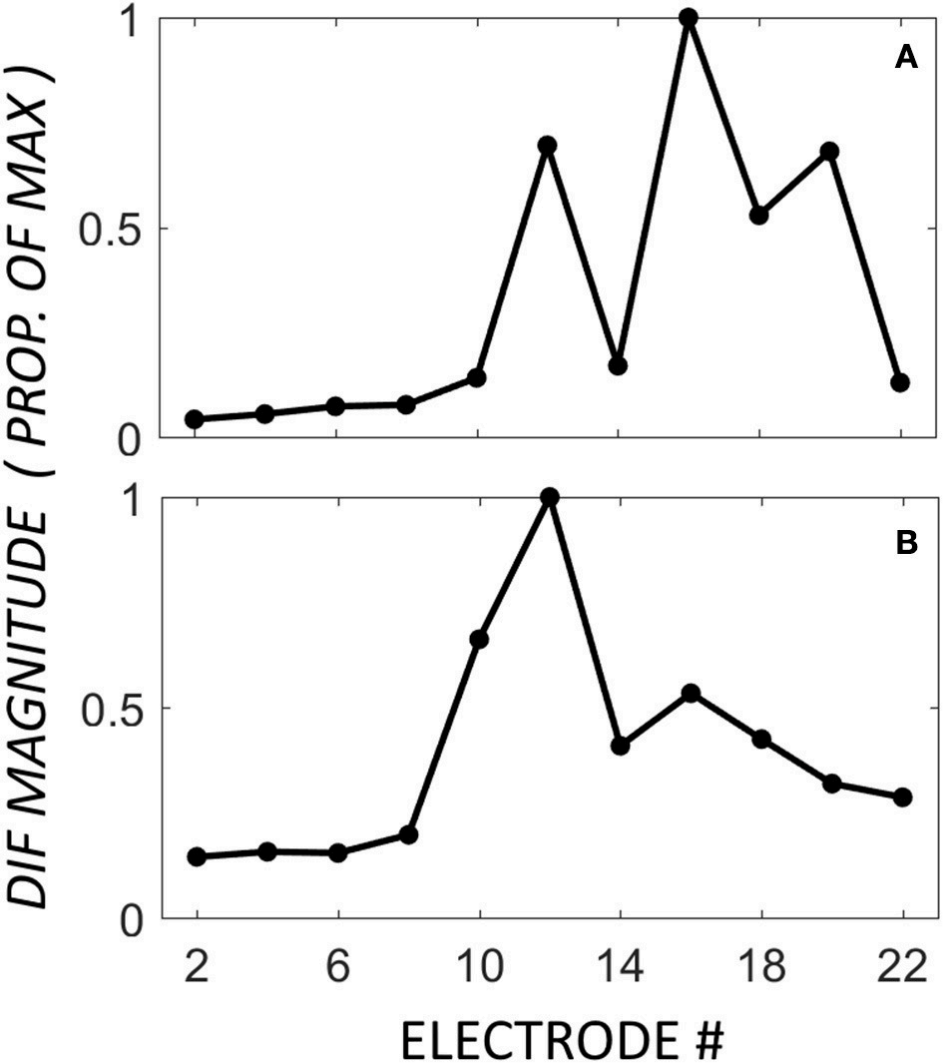
**DIF amplitude across the electrode array in two individuals from the mid-electrode peak group**. In these individuals, the mid-electrode peaks were on electrode 12. For **(A)**, the DIF amplitude increased to a second peak on an apical electrode (16). For **(B)**, the DIF amplitude decreases across the final 5 recording electrodes.

A third, previously undescribed, pattern showed a mid-electrode peak of DIF amplitude with or without a second apical peak (*n* = 8). The mid-electrode peaks occurred most frequently on electrode 12 (*n* = 5), and less frequently on electrodes 14 (*n* = 2), and 8 (*n* = 1). Apical to the mid-electrode peaks, the DIF amplitude increased to a second peak on apical electrodes in half of the participants in this group (*n* = 4), and decrease to a flat response in half (*n* = 4). Examples of these patterns are shown in Figure 4, demonstrating a second apical peak (Figure 4A) or a single mid-electrode peak (Figure 4B).

**Figure 5:**
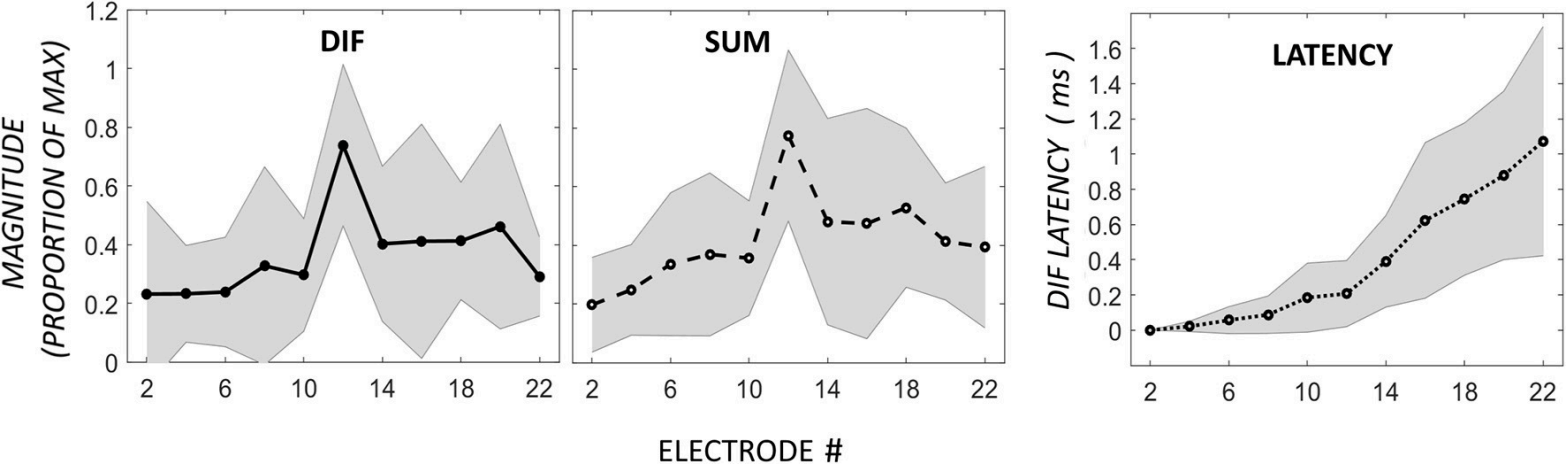
**DIF, SUM, and DIF latency responses in 8 participants with a mid-electrode peak, for electrodes 2–22**.

**Figure 6:**
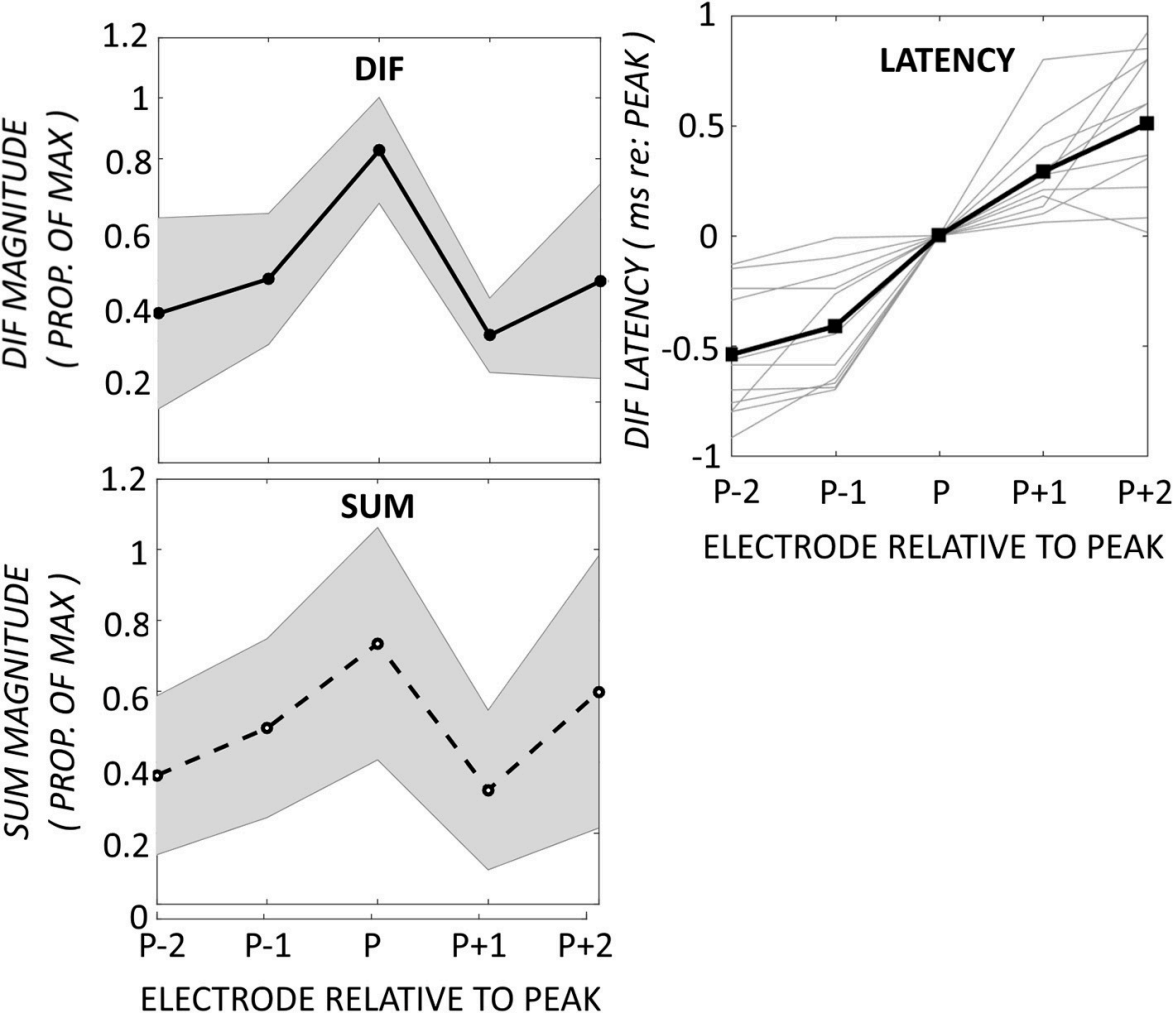
**Changes in DIF and SUM amplitudes and DIF latencies have been aligned to the electrode exhibiting the mid-electrode peak (P)**. The DIF and SUM responses show peaks on the same electrodes. The DIF latency increased progressively across the peak. Shaded area represents ± 1 *SD*.

Figure 5 demonstrates the normalized DIF and SUM magnitudes, and the DIF latencies, that have been averaged across the patients for each of the electrodes in the mid-electrode peak group. The DIF amplitudes at these mid-electrode peaks (27.7 μV ± 10.4 SEM) were comparable to the maximal amplitudes seen in the apical-peak group. The latency increased from the most basal electrode (1.0-ms ±.52) to a mean of 2.55-ms ±.70 at the tip of the electrode. Because the electrode upon which the peak occurred varied between subjects, these data were replotted, but now referenced to the mid-electrode peak for each individual (Figure 6). By aligning these peaks, it is apparent that the SUM amplitude peaks on the same electrode as the DIF amplitude. In addition, latency grew progressively across the peak.

As the ECochG signal is comprised of potentials derived from both neural and sensory elements, one possible explanation for the mid-electrode peaks was that these were generated by constructive or destructive interference between the phases of these potentials. If this were the case, it would be expected that the phase of the DIF and SUM components would be constructive at the peak electrode. In contrast to this expectation, there was no consistent relationship between the phase of the DIF and SUM components at the peak, or the surrounding electrodes. The difference in phase between peak electrode and the next most basal electrode averaged −1.7° ± 16, and between peak electrode and next most apical the phase difference was −15.7° ± 41, which is not consistent with an advancement from destructive to constructive interference between the CM and ANN on the mid-electrode peaks.

### Patterns relationship to hearing level

**Figure 7:**
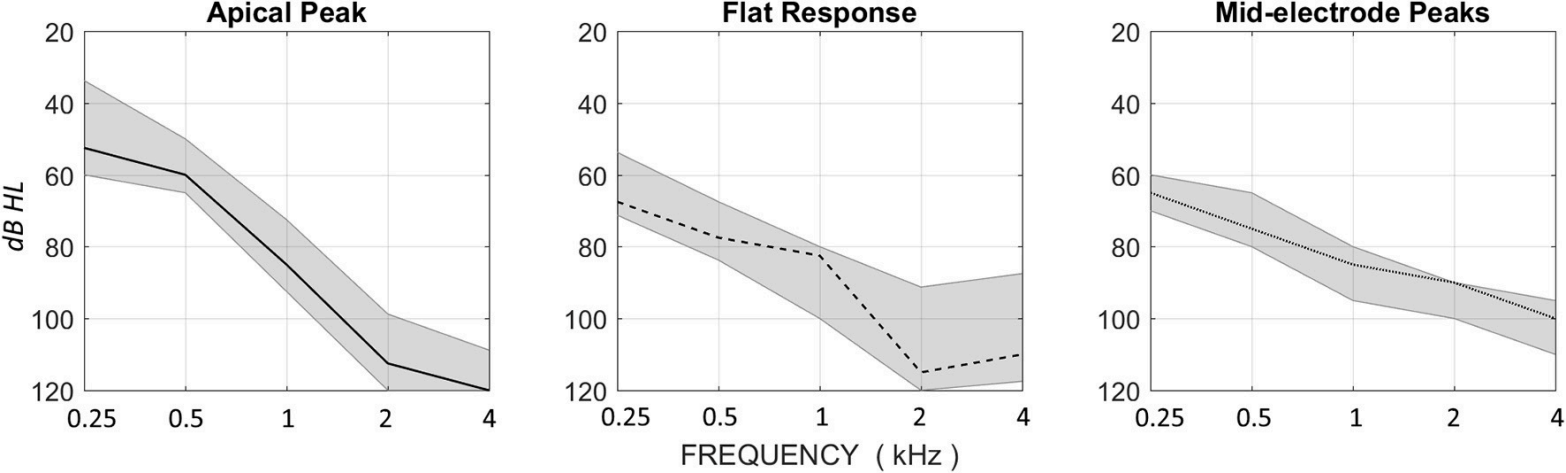
**Median HL for the Apical Peak, Flat Response, and Mid-electrode Peak groups, with upper and lower quartile ranges**. The Apical Peak group has the best low-frequency thresholds, and the Mid-electrode Peak group has the best high-frequency thresholds. The Flat Response group has poor thresholds across the audiometric range.

Figure 7 summarizes the audiometric results for each of the three response patterns. Audiometric thresholds at 0.5-kHz were significantly lower in the apical peak group than the other two groups [Kruskal–Wallis test, H(2) = 7.43, *p* = 0.024]. The flat-response group showed the highest level of low-frequency hearing loss. The mid-electrode peak group showed the lowest level of high-frequency hearing loss and peaks in DIF amplitude that were at cochlear locations proximal to these high-frequency regions in the cochlea.

## Discussion

Here we describe three different response patterns, characterized as the response to a high intensity 0.5-kHz acoustic stimulus, when ECochG was recorded along the length of a cochlear electrode immediately after surgical implantation of the array. All patients had residual hearing recorded on their pre-operative audiograms.

For ease of communication the term CM will be used to refer to the first harmonic of the DIF response, and the ANN to the second harmonic of the SUM response. It is acknowledged that other cochlear generators may have contributed to these responses, such as a neural response to the first harmonic of the DIF response (Forgues et al., 2014), and hair cell distortion products to the second harmonic of the SUM (Teich et al., 1989), but these are of smaller magnitude and do not impact significantly upon the response characterization proposed here.

The apical response pattern that was expected from cochleae with functioning hair cells in the more apical cochlear regions. This is supported by the growth of CM amplitude along the length of the cochlea, and by the relatively low audiometric thresholds at 0.25 and 0.5-kHz. In these patients hearing dropped to a median of 85-dB at 1-kHz, and to profound levels above this. The latency of the CM is also consistent with this interpretation, as it became more prolonged toward the tip of the electrode, especially in responses recorded from electrodes in the apical half of the array where the response amplitude was growing more rapidly. This is what might be expected if the ECochG recorded from each electrode reflected the response of local populations of hair cells, in response to a cochlear traveling wave generated by a 0.5-kHz tone. Further support for this notion comes from the latency growth observed along the electrode, which was similar—but slightly shorter than—that seen in human psychophysical experiments for a traveling wave traversing this region of the cochlea (Eggermont, 1979; Schoonhoven et al., 2001). The shorter latency may reflect a basal-ward shift in the cochlear site of excitation arising from the high intensity of the acoustic stimulus (Honrubia and Ward, 1968; Russell and Nilsen, 1997). The peak CM amplitude occurred a few electrodes away from the tip in some patients, and dropped dramatically in magnitude on the more apical electrodes. This we suspect is a result of the tip of the electrode passing the 0.5-kHz place on the basilar membrane. Alternatively, this response characteristic might have been caused by the most apical implant electrodes contacting the basilar membrane, as this would prevent motion of the basilar membrane and dampen hearing at the point(s) of contact. The ANN response amplitude correlated well with the CM in this group of patients, presumably reflecting good innervation of the residual hair cells. However, the electrode upon which the CM and ANN peaks occurred differed in more than half the patients, usually with the CM peak on a more basal electrode. The reason(s) for this discrepancy are not apparent.

The CM latency for the mid-electrode peak group resembled that of the apical peak responders, as is apparent in Figures 4, 5. This suggests that the mid-electrode peak may arise from surviving populations of hair cells in more basal regions of the cochlea, as this electrode is typically located around 10-mm into the cochlea, near the 2-kHz region, and this group had the best audiometric thresholds at 2-kHz (90 dB HL in the mid-electrode peak group, compared with 112.5 or 115 in the Apical Peak or Flat Response groups, respectively). An alternative explanation for a mid-electrode peak might be the constructive interference of the phases of CM and ANN, but there was no evidence to support this in the data presented. Our results suggest that those hair cells which are present are likely to be innervated, as the profile of the ANN response mirrors that of the CM response.

The flat ECochG response pattern was also found in Dalbert et al. (2015) and Calloway et al. (2014). This pattern occurred in the individuals with higher levels of hearing loss than the other two groups, with median audiometric thresholds that were 15-dB worse at 0.25-kHz and 17.5-dB at 0.5-kHz than those in the apical peak group. Thresholds in response to higher frequencies were similar to those seen in patients exhibiting an apical response. The very slow CM amplitude growth across the electrode, with a long latency response (>2 ms) that changed little across the electrode suggests that the response detected arose from the apex of the cochlea, and that hair cell responses were not detected in the vicinity of the implant's electrodes. In addition, it might be that with the poorer hearing seen in these subjects, our system did not have sufficient acoustic drive to elicit a robust response. Based on these findings, it is suggested that flat responders reflect cochleae with “dead regions,” namely cochlear places without significant numbers of functioning hair cells. The identification of dead regions is of clinical significance, as they will limit any benefit of EAS.

The present work identified discrete patterns of ECochG profile across the electrode that related to the patient's residual hearing. By recording ECochG across the electrode array, it was possible to map out the location of functioning hair cells and infer whether these were innervated. These data may improve the fitting of electro-acoustic hearing aids in the future, as specific regions with hair cell survival can be targeted with the acoustic component. The approach provides a detailed assessment of cochlear health at the time of cochlear implantation that provides a baseline for longitudinal monitoring of residual hearing. It is hoped that this will provide unique insights into the nature of hearing loss in the months after implant surgery. Furthermore, as these responses are better characterized, it will be possible to correlate the ECochG profile with speech perception and determine whether particular cochlear pathologies predict the outcome of cochlear implantation.

## Author contributions

CB and SO: Design of study, data collection and analysis, writing and proofreading of manuscript. LC: Design of study, data collection and analysis, and proofreading of manuscript. AD: Data collection and analysis, and proofreading of manuscript. AC: Data analysis and proofreading of manuscript.

## Funding

SO was funded by the National Health and Medical Research Council (Australia), GNT0628679 and GNT1078673.

### Conflict of interest statement

It is disclosed that the University of Melbourne is supported by research funding from Cochlear Ltd. The authors declare that the research was conducted in the absence of any commercial or financial relationships that could be construed as a potential conflict of interest.
